# Efficacy and Safety of Tenofovir Disoproxil Orotate in Chronic Hepatitis B Patients Previously Treated with Tenofovir Disoproxil Fumarate: Multicenter, Open-Label, Prospective Study

**DOI:** 10.3390/jcm10235628

**Published:** 2021-11-29

**Authors:** Young Chang, Sang-Gyune Kim, Soung-Won Jeong, Jae-Young Jang, Jeong-Ju Yoo, Sae-Hwan Lee, Young-Seok Kim, Hong-Soo Kim, Hyun-Woong Lee, Suyeon Park

**Affiliations:** 1 Digestive Disease Center, Department of Internal Medicine, Institute for Digestive Research, Soonchunhyang University College of Medicine, Seoul 04401, Korea; chyoung@schmc.ac.kr (Y.C.); jyjang@schmc.ac.kr (J.-Y.J.); 2 Department of Internal Medicine, Soonchunhyang University College of Medicine, Bucheon 14584, Korea; mcnulty@schmc.ac.kr (S.-G.K.); puby17@schmc.ac.kr (J.-J.Y.); liverkys@schmc.ac.kr (Y.-S.K.); 3 Department of Internal Medicine, Soonchunhyang University College of Medicine, Cheonan 31151, Korea; stevesh@schmc.ac.kr (S.-H.L.); khskhs@schmc.ac.kr (H.-S.K.); 4 Department of Internal Medicine, Gangnam Severance Hospital, Yonsei University College of Medicine, Seoul 06273, Korea; 5 Department of Biostatistics, Soonchunhyang University Hospital, Seoul 04401, Korea; suyeon1002@schmc.ac.kr; 6 Department of Applied Statistics, Chung-Ang University, Seoul 06974, Korea

**Keywords:** chronic hepatitis B, tenofovir disoproxil orotate, noninferiority, HBcrAg, renal function

## Abstract

Background/Aim: We aimed to demonstrate the efficacy and safety of tenofovir disoproxilorotate (TDO) compared with that of tenofovir disoproxil fumarate (TDF) in patients with chronic hepatitis B. Methods: This multicenter, open-label, prospective clinical trial (KCT0004185) was conducted to evaluate the efficacy and safety of TDO on switching from TDF for 24 weeks in virologically suppressed chronic hepatitis B patients. The primary efficacy endpoint was the maintenance of virologic response. Safety was assessed by evaluating major adverse events, changes in renal function, and occurrence of hepatocellular carcinoma (HCC). Results: TDO treatment was not inferior in terms of virological response when compared with that on TDF treatment, with a noninferiority margin of −10% (risk difference, −3.17%; 95% confidence interval, −7.5–1.15%). The biological response of TDO was also comparable to that of TDF, with no significant difference in the proportion of patients with normalized alanine transaminase levels. After 24 weeks of treatment, hepatitis B core-related antigen (HBcrAg) significantly decreased to a mean titer of 3.91 log U/mL from 4.15 log U/mL at baseline (*p* = 0.01). There were no cases of grade 3 or higher adverse events and HCC. The mean estimated glomerular filtration rate increased from 91.09 mL/min to 93.34 mL/min (*p* = 0.056), and the mean serum level of phosphorus increased from 3.33 mg/dL to 3.44 mg/dL (*p* = 0.045), suggesting improvement in renal function with TDO treatment. Conclusion: In patients with chronic hepatitis B, the efficacy of TDO was noninferior to that of TDF, with a significant decrease in the HBcrAg titer and improved renal function.

## 1. Introduction

Chronic hepatitis B is a major global health concern. In 2017, the World Health Organization estimated that 257 million people have suffered from the infection, resulting in approximately 887,000 deaths, mostly from cirrhosis and hepatocellular carcinoma [1]. Antiviral treatment with nucleos(t)ide analogs (NA) can delay liver disease progression and decrease liver-related complications [2,3,4]. Unfortunately, the functional cure for chronic hepatitis B, defined by the loss of hepatitis B surface antigen (HBsAg), with NA treatment is uncommon [5], and hepatitis B viral reactivation occurs frequently when NA treatment is discontinued [6]. Therefore, a long-term antiviral treatment is required for most patients with chronic hepatitis B. However, long-term NA treatment is expensive and can result in drug resistance and adverse events.

Tenofovir is a nucleotide analog reverse transcriptase inhibitor that terminates viral replication by inhibiting viral DNA polymerases. Tenofovir disoproxil, a prodrug form of tenofovir, was developed to improve its bioavailability because tenofovir exists in a highly polar dianion state at physiological pH [7]. To increase solubility, tenofovir disoproxil was conjugated with fumaric acid to produce tenofovir disoproxil fumarate (TDF) [8]. TDF is an orally bioavailable NA, approved in 2008, for the first-line treatment of chronic hepatitis B, owing to its potent antiviral effect and high genetic barriers against viral resistance [9].

Orotates are widely used in conjugation with other active ingredients. A novel orotate form of tenofovir disoproxil (tenofovir disoproxil orotate (TDO)) was developed to reduce manufacturing costs and ultimately provide a cost-effective treatment for patients with chronic hepatitis B. A comparative study of pharmacokinetic analysis has demonstrated that the serum tenofovir concentration-time profiles of TDF and TDO were comparable, with similar maximum concentration (C_max_), time to reach C_max_, and area under the concentration–time curve [10]. In addition, there were no clinically significant findings in the tolerability assessments after TDF and TDO administration. Based on these bioequivalence test results, TDO, a generic drug of TDF, was approved by the Korean Ministry of Food and Drug Safety as a treatment for chronic hepatitis B.

Through this clinical trial, we aimed to compare the antiviral efficacy and safety of TDO and TDF in patients with chronic hepatitis B after a 24-week treatment period.

## 2. Methods

### 2.1. Study Population

In this multicenter study, 63 patients were prospectively enrolled from four representative academic hospitals in Korea according to the following criteria: were >19 years and had received TDF monotherapy for chronic hepatitis B for at least 48 weeks. The patients were also required to have suppressed HBV DNA levels <20 IU/mL and be willing to voluntarily participate in the study with written consent. Patients coinfected with hepatitis C, hepatitis D, or human immunodeficiency virus; those with evidence of decompensation (i.e., ascites, encephalopathy, or variceal hemorrhage), liver transplant recipients, and those with hepatocellular carcinoma (HCC) were excluded. Patients with impaired renal function, defined as having creatinine clearance less than 50 mL/min (by the Cockcroft–Gault method), were also excluded.

### 2.2. Study Design

This was a multicenter, open-label, investigator-initiated, prospective study to evaluate the efficacy and safety of TDO compared with TDF in chronic hepatitis B patients. Eligible patients with suppressed HBV DNA under TDF treatment were replaced with TDO (319 mg per tablet once daily) for 24 weeks. After initiating treatment, the patients visited the hospital every 12 weeks to undergo clinical and laboratory examinations and completed 24 weeks of treatment unless there was a reason for discontinuation of medical examination and drug prescription. At every visit, patients underwent a physical examination, vital sign check, and laboratory examinations, including complete blood count, chemistry, and coagulation tests.

### 2.3. Laboratory Assays

Serum samples collected at the index date and stored at Soonchunhyang University Seoul Hospital were prospectively studied. Hepatitis B core-related antigen (HBcrAg) level was measured by chemiluminescence immunoassay on a LUMIPULSE G12000 automated analyzer (Fujirebio, Tokyo, Japan) at Gangnam Severance Hospital, Seoul, following the manufacturer’s instructions. The range of HBcrAg quantification was 3.0 to 7.0 log U/mL. Samples with an HBcrAg level above 7.0 log U/mL were diluted and retested to measure the HBcrAg level.

### 2.4. Endpoints

The primary efficacy endpoint was the maintenance of the virologic response rate, defined as the proportion of patients with HBV DNA level <20 IU/mL after 24 weeks of TDO treatment. The secondary efficacy endpoints were the proportion of normalized serum alanine transaminase (ALT) levels at each visit, hepatitis B e antigen (HBeAg) loss rate, and changes in HBcrAg titer at the end of 24-week TDO treatment. The descriptive statistics of the change in HBV DNA titer from baseline were evaluated at each visit.

Major adverse events, renal parameters including estimated glomerular filtration rate (eGFR) and serum phosphorus level, and occurrence of HCC were evaluated for safety assessment.

### 2.5. Statistical Analyses

Since the probability of achieving an HBV DNA level of less than 20 IU/mL at 48 weeks on maintenance of TDF was assumed to be 93% with reference to previous studies [11,12], the noninferiority margin was assumed to be 10%. Accordingly, at least 52 patients were required to demonstrate that the proportion of patients with a maintained virologic response after administration of TDO for 24 weeks was not inferior to that resulting from administration of TDF. Assuming a dropout rate of 20%, the ideal total number of the study population was calculated to be 64.

For primary efficacy endpoint analysis, the proportion of subjects with HBV DNA level <20 IU/mL at Week 24 was calculated, and a one-sided 95% confidence interval for the difference in this proportion compared to that at baseline was estimated. If the lower limit of this estimated confidence interval was greater than the preset noninferiority limit of −10%, TDO was evaluated as noninferior compared to TDF.

Paired nominal variables were compared using McNemar’s test, and paired continuous variables were compared using the paired sample *t*-test or Wilcoxon signed-rank test. For independent variables, a two-sample *t*-test or Wilcoxon rank-sum test, and for categorical variables, χ^2^-test or Fisher’s exact test was used.

### 2.6. Ethical Declaration

This study was conducted in four institutions, and signed informed consent was submitted by all participants; the study protocol conformed to the ethical guidelines of the World Medical Association Declaration of Helsinki. This study was approved by the Institutional Review Board of Soonchunhyang University Seoul Hospital (Number 2019-04-012).

## 3. Results

### 3.1. Baseline Characteristics of the Study Population

A total of 67 patients with chronic hepatitis B were screened from September 2019 to June 2020; three did not meet the inclusion criteria, and one dropped out after withdrawal of consent. Finally, 63 patients were prospectively enrolled in this clinical trial. Table 1 presents the baseline characteristics of the study population. The mean age was 49 years, and 65.6% were males. The median levels of serum aspartate transaminase (AST) and ALT were within the normal range. The mean serum creatinine level was 0.89 mg/dL, resulting in a mean estimated glomerular filtration rate (eGFR) of 91.28 mL/min, and a median serum phosphorus level of 3.3 mg/dL. HBeAg was positive in 27% (17 of 63) of the study population, and the mean serum HBcrAg titer was measured as 4.25 log U/mL.

### 3.2. Virologic Response

Primary efficacy assessment was performed by comparing the proportion of patients with suppressed viral replication (<20 IU/mL) at baseline and 24 weeks after TDO treatment. At 12 weeks of TDO administration, the whole study population maintained suppressed HBV DNA levels of <20 IU/mL. After 24 weeks of treatment, 96.83% (61 of 63) of patients maintained a virologic response with HBV DNA levels <20 IU/mL, and two patients exhibited HBV DNA levels of 21.1 IU/mL and 35.8 IU/mL, respectively. There was no statistical difference in the proportion of patients with viral suppression compared to the baseline (100% vs. 96.83%; *p* = 0.5), and TDO treatment was noninferior compared with TDF treatment in terms of virologic response, with a noninferiority margin of −10% (risk difference, −3.17%; 95% confidence interval, −7.5–1.15%).

In a subgroup analysis based on HBeAg status at baseline, all the HBeAg-negative patients (*n* = 46) maintained virologic response at Week 24, whereas 88.2% of HBeAg-positive patients (15 of 17) maintained a virologic response.

### 3.3. Biochemical and Serologic Responses

**Figure 1:**
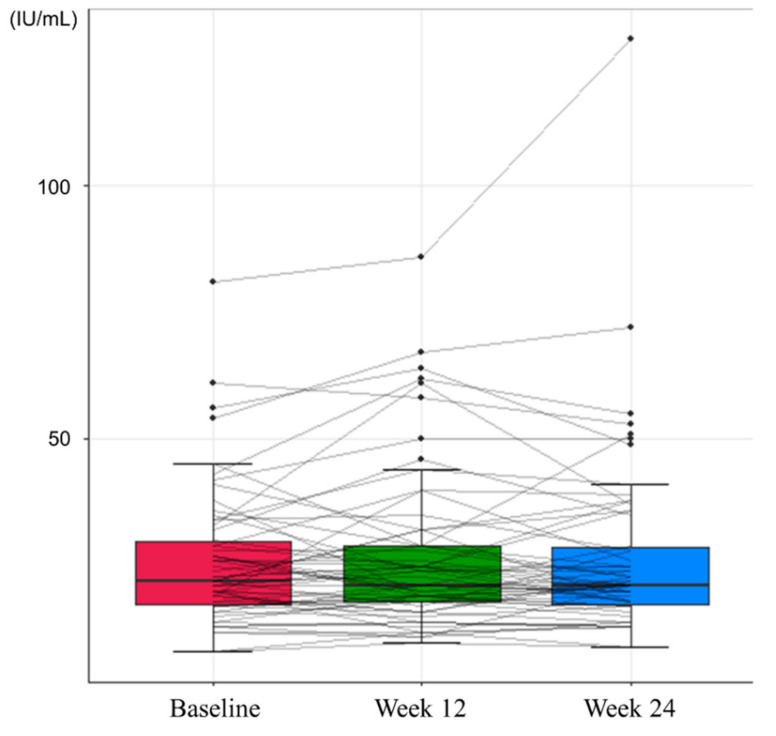
Changes in serum alanine transaminase levels during the study period.

The proportions of patients with normal levels of serum ALT were 87.3% at baseline, 82.26% at Week 12, and 87.3% at Week 24, indicating no significant differences between baseline and Weeks 12 and 24 (*p* = 0.45 and 1.00, respectively; Table 2). The mean levels of ALT at baseline, Week 12, and Week 24 were 25.63 IU/mL, 26.92 IU/mL, and 26.75 IU/mL, respectively, with no significant differences between groups (Figure 1).

**Figure 2:**
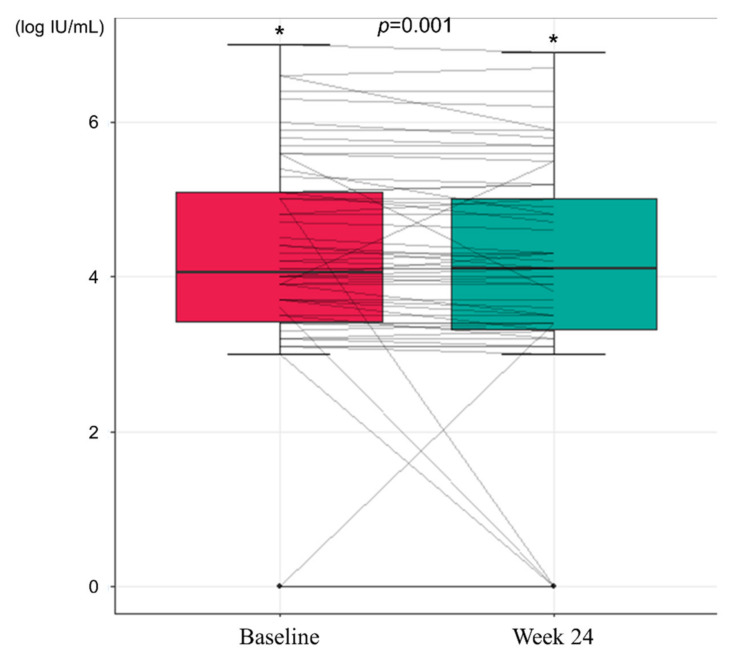
Changes in hepatitis B core-related antigen levels during the study period (* *p* < 0.05).

During the study period, there was no HBeAg loss or seroconversion. However, there was a significant decrease in HBcrAg titer after 24 weeks of treatment with TDO. At baseline, the mean HBcrAg titer was 4.154 log IU/mL, which significantly decreased to 3.915 log IU/mL at Week 24 (*p* = 0.001, Figure 2).

### 3.4. Changes in HBV DNA Titer

There was no virological breakthrough during the study period. Among the 63 patients, four exhibited an increased HBV DNA titer at 24 weeks of treatment from the baseline. One patient had an HBV DNA level <20 IU/mL (detection limit of the institution) at baseline, which increased to 21.1 IU/mL at Week 24. The other three patients had HBV DNA levels <15 IU/mL (detection limit of the institution) at baseline, which rose to 15.5 IU/mL, 19.1 IU/mL, and 35.8 IU/mL at Week 24. In all four cases, HBV DNA titer was found to be below the detection limit of each institution (20 or 15 IU/mL) at the follow-up examination after the end of the study.

### 3.5. Safety Assessment

**Figure 3:**
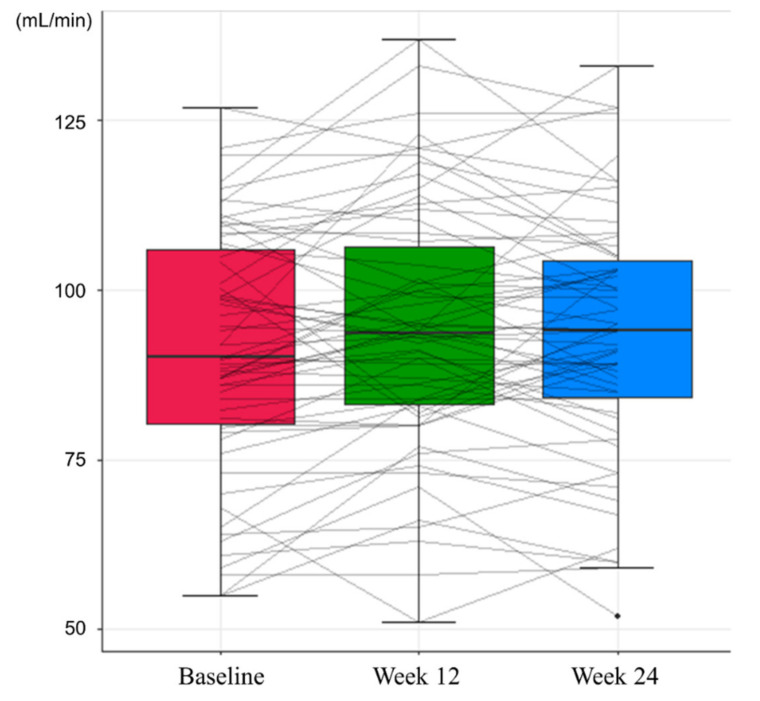
Changes in estimated glomerular filtration rates during the study period.

**Figure 4:**
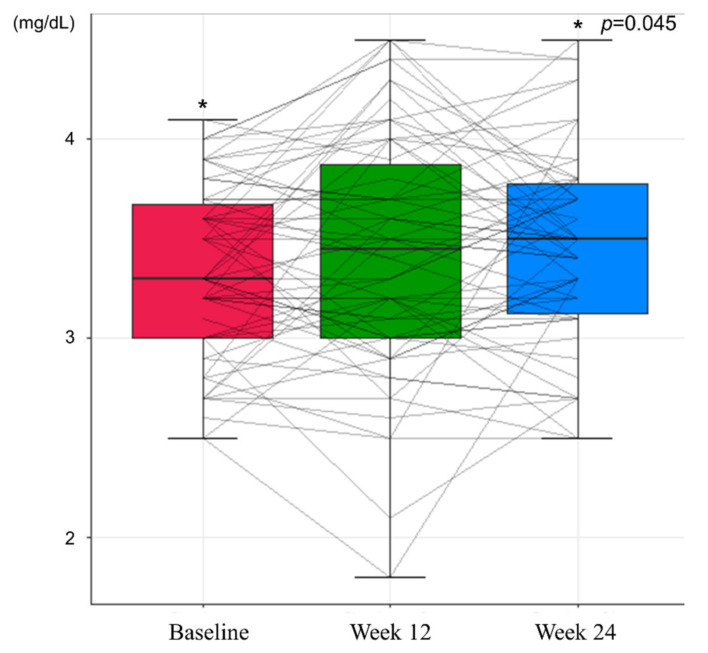
Changes in serum phosphorus levels during the study period (* *p* <0.05).

In the safety assessment, there were no cases of grade 3 or higher adverse events and HCC during the study period. At Week 12, no adverse events were observed. Grade 1 general weakness and lower abdominal pain were reported at Week 24, which recovered spontaneously. Renal function, assessed by eGFR and serum phosphorus level, showed an improving trend during the study period (Table 3). Mean eGFR of 91.09 mL/min at baseline increased to 93.34 mL/min at Week 24 (*p* = 0.056, Figure 3), and the mean serum level of phosphorus increased from 3.33 mg/dL to 3.44 mg/dL at 24 weeks (*p* = 0.045, Figure 4). While the proportion of patients with renal dysfunction was 1.59% due to a patient whose serum phosphorous level decreased below 2 mg/dL at Week 12, the patient recovered spontaneously, resulting in 0% of patients with renal dysfunction at Week 24.

## 4. Discussion

This prospective study of administering TDO for the treatment of chronic hepatitis B demonstrated the noninferiority of TDO to TDF in the efficacy of viral suppression at Week 24. TDO treatment showed maintained virologic and biochemical responses comparable with that on TDF treatment. No viral breakthrough occurred during TDO treatment; moreover, the HBcrAg titer significantly decreased after 24-week treatment with TDO. TDO showed a better safety profile than TDF in terms of renal parameters, and none of the patients discontinued its use due to adverse events.

According to recent clinical practice guidelines, various antiviral agents recommended as first-line antiviral agents show a variable virologic response rate of 64–76% in HBeAg-positive patients and 90–94% in HBeAg-negative patients [13,14]. In the current study, patients with positive HBeAg showed a lower rate of virologic response than those with negative HBeAg, in accordance with previous study results. The proportion of patients with maintained virologic response after 24 weeks of TDO treatment was 88.2% among HBeAg-positive patients and 100% among HBeAg-negative patients. Two patients failed to maintain a virologic response, with an HBV DNA level of 21.1 IU/mL and 35.8 IU/mL at Week 24 without biochemical aggravation, and they spontaneously had HBV DNA titers below the detection limit of each institution at follow-up after the study was completed, suggesting that the antiviral efficacy of TDO is comparable with that of TDF.

HBeAg loss or seroconversion rates were lower than those reported in previous studies that reported 8–14% after a 48-week treatment [15] and 28.3–41.7% after a 5-year treatment [3,16]. Although HBV genotype was not identified in individual participants in this study, it can be assumed that most of the study population had genotype C HBV considering the geographic distribution [17,18], which was reported to have lower rates of HBeAg seroconversion than other genotypes [19]. The relatively short duration of antiviral treatment may also have contributed to the low seroconversion rate.

In contrast, TDO treatment induced a significant decrease in the HBcrAg titer despite the short treatment period. HBcrAg consists of three proteins: hepatitis B core antigen (HBcAg), HBeAg, and 22-kDa precore protein (p22cr) [20]. HBcAg is a nucleocapsid surrounding HBV DNA, and HBeAg is a circulating protein derived from the core gene that serves as a marker of viral replication activity [21]. The HBcrAg titer correlates well with the levels of HBV DNA and covalently closed circular DNA (cccDNA) [22,23,24]. Wong et al. reported that HBcrAg correlates positively with cccDNA in patients with detectable HBV DNA, as well as in patients who achieved viral suppression with antiviral therapy [24]. HBcrAg is a surrogate marker for cccDNA and its transcriptional activity [25]. Furthermore, several studies have reported the role of HBcrAg in predicting HCC development during antiviral therapy. Patients with higher serum HBcrAg levels were associated with an increased risk of HCC, even when HBV DNA was undetectable with antiviral therapy [26]. Serum levels of HBcrAg in the current study were relatively low, with a mean titer of 4.15 log IU/mL, because all the patients achieved viral suppression with sufficient periods of antiviral therapy at baseline. In this study population, TDO treatment further decreased the HBcrAg level to 3.92 log IU/mL, which can have a protective effect on HCC.

Renal toxicity is a concern in chronic hepatitis B patients receiving long-term TDF treatment [27]. Renal toxicity of TDF is indicated by decreased GFR and renal tubular injury [28]. In the present study, eGFR and serum phosphorus levels increased with marginal statistical significance after switching from TDF to TDO, suggesting that TDO has a renal protective effect when compared to TDF. Thus, TDO can be safely used in patients treated with TDF without further deterioration of renal function.

TDO has an antiviral effect comparable to TDF and at the same time has several advantages over TDF. Careful attention should be paid to the surrounding climate during the manufacture and storage of TDF because of its low thermo- and photostability. In addition, its hygroscopicity further causes difficulties in manufacturing control (WO2015002434A1). TDO has improved stability over TDF, prolonging its shelf life. Furthermore, TDO can lower the cost involved in the manufacture of salt conjugates compared to TDF [10]. These advantages of TDO can ultimately improve patients’ quality of life.

There are several limitations in the present study. First, the study population consisted of only Asians, predominantly with HBV genotype C, due to the nature of the study design. The sample size of the study population was relatively small, and HBeAg positivity was not evenly distributed among the study population, because we set the minimum sample size that could be statistically proven effective. Further large-scale, ethnically diverse clinical trials with various HBV genotypes are required to validate the efficacy and safety of TDO. Second, it is still unclear why TDO exhibited renal protective effects compared to TDF, as a previous study reported that TDF and TDO exhibited similar pharmacokinetics [10]. Moreover, since both GFR and *p* values were changed within the normal range in each group, the clinical significance may be limited. Third, we did not evaluate bone mineral density due to the short treatment duration. Additionally, since the study period is relatively short, long-term efficacy and safety evaluation are necessary. In the same context, although there are significant short-term improvements in renal outcomes, it is unclear whether these differences persist during long-term treatment with TDO. Long-term follow-up studies are required in the future. Despite these limitations, to the best of our knowledge, this is the first study to report the efficacy and safety of TDO therapy.

In conclusion, the antiviral efficacy of TDO is comparable to that of TDF, with favorable renal outcomes. TDO can be a suitable candidate in terms of efficacy, safety, and economics as an alternative to TDF in patients with chronic hepatitis B.

## Figures and Tables

**Table 1:** Baseline characteristics of the study population.

	Total (*n* = 63)
Age (year)	49.05 ± 10.93
Male, no. (%)	42 (65.62%)
Platelet count	204.34 ± 56.03
Albumin (g/dL)	4.7 (4.5, 4.9)
Total bilirubin (mg/dL)	0.76 (0.5, 0.95)
Aspartate transaminase (U/L)	22 (19.75, 28)
Alanine transaminase (U/L)	22 (17, 30.5)
Prothrombin time (INR)	1.04 (1, 1.07)
Creatinine (mg/dL)	0.89 ± 0.17
Glomerular filtration rate (ml/min)	91.28 ± 17.58
Phosphorus (mg/dL)	3.3 (3, 3.62)
HBeAg positive, no. (%)	17 (26.98%)
HBcrAg (log U/mL)	4.15 ± 1.61
Diabetes, no. (%)	6 (9.38%)
Hypertension, no. (%)	10 (15.62%)

**Table 2:** Biochemical response of TDO treatment at Weeks 12 and 24.

	ALT (IU/mL)	*p* Value	Proportion of Normal ALT (%)	*p* Value
Baseline	25.63 ± 13.24	Ref.	87.3%	Ref.
Week 12	26.92 ± 15.84	0.285	82.26%	0.453
Week 24	26.75 ± 18.10	0.387	87.3%	1.000

TDO, tenofovir disoproxil orotate; ALT, alanine transaminase.

**Table 3:** Changes in renal function after TDO treatment during the study period.

	eGFR (IU/mL)	*p* Value	Phosphorus (mg/dL)	*p* Value
Baseline	91.09 ± 17.66	Ref.	3.33 ± 0.43	Ref.
Week 12	94.46 ± 18.26	0.010	3.43 ± 0.60	0.119
Week 24	93.34 ± 17.80	0.056	3.44 ± 0.49	0.045

TDO, tenofovir disoproxil orotate; eGFR, estimated glomerular filtration rate.

## Data Availability

The data presented in this study are available on request from the corresponding author. The data are not publicly available due to privacy.
